# A rare case of Krukenberg tumor by gallbladder cancer

**DOI:** 10.1016/j.amsu.2019.09.014

**Published:** 2019-10-04

**Authors:** Antonio Pesce, Giovanni Li Destri, Francesca Flavia Amore, Gaetano Magro, Gaetano La Greca, Stefano Puleo

**Affiliations:** Department of Medical and Surgical Sciences and Advanced Technologies “G.F. Ingrassia”, University of Catania, Italy

**Keywords:** Case report, Gallbladder cancer, Uncommon finding, Metastatic localization, Ovarian mass, Krukenberg tumor

## Abstract

**Introduction:**

Gallbladder cancer commonly spreads by direct extension to the liver and adjacent organs of the gastrointestinal tract. Ovarian metastases by biliary origin, though known, are a very uncommon finding.

**Presentation of case:**

We report a rare metastatic localization by gallbladder cancer, Krukenberg tumor mimicking a primitive ovarian cancer. A comprehensive critical review was performed and suggested strategies were analyzed.

**Discussion:**

The prognosis of ovarian metastastes by biliary origin is very poor with an overall survival estimated at around 6 months. The variable clinical presentation, radiology and serum markers make the appropriate histological diagnosis mandatory.

**Conclusion:**

The presence of Krukenberg tumor should be considered in the work-up of gallbladder cancer.

## Highlights

• Gallbladder carcinoma is a rare malignancy with a significant source of mortality in selected areas of high incidence.
• Krukenberg tumor by gallbladder cancer is a very uncommon finding with diagnostic difficulties for clinicians.
• The variable clinical presentation, radiology and serum markers make the appropriate histological diagnosis mandatory.
• The presence of Krukenberg tumor should be considered in the work-up of gallbladder cancer.

## 1 Introduction

According to GLOBOCAN 2018 data, gallbladder cancer is the 22nd most incident but 17th most deadly cancer worldwide. In 2018, about 219 000 people were estimated to have been diagnosed with gallbladder cancer [1,2]. In selected areas of high incidence, such as India, Chile and Japan, it represents a significant source of mortality, unlike most Western countries where the incidence is very low [3].

However, 1–2% of surgical specimens demonstrated a gallbladder cancer as an incidental finding [2,3]. Most gallbladder cancers are adenocarcinoma (approximately 70–90%) and they are usually confined to the gallbladder and the adjacent liver [2]. This cancer commonly spreads by direct extension to the liver and adjacent organs of the gastrointestinal tract. Gallbladder cancer with ovary spread is quite rare with only few reports available in the English literature [[4], [5], [6], [7], [8]].

Here, the authors describe an uncommon clinical presentation of gallbladder cancer with the presence of Krukenberg tumor by biliary origin, mimicking a primitive ovarian cancer in an academic medical center.

## 2 Methods

The work has been reported in line with the SCARE 2018 criteria [9]. However, this case report is not the “*first case in man*”, neither a new device or surgical technique was performed; for this reason, it was exempt from registering into the Research Registry.

## 3 Case report

**Fig. 1:**
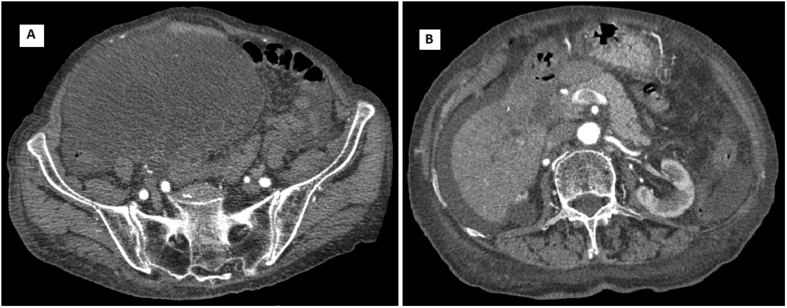
Abdominal CT scan with axial views revealed a voluminous mass in the right iliac fossa (A), the presence of ascites and a thick-walled gallbladder adherent to the duodenum (B).

**Fig. 2:**
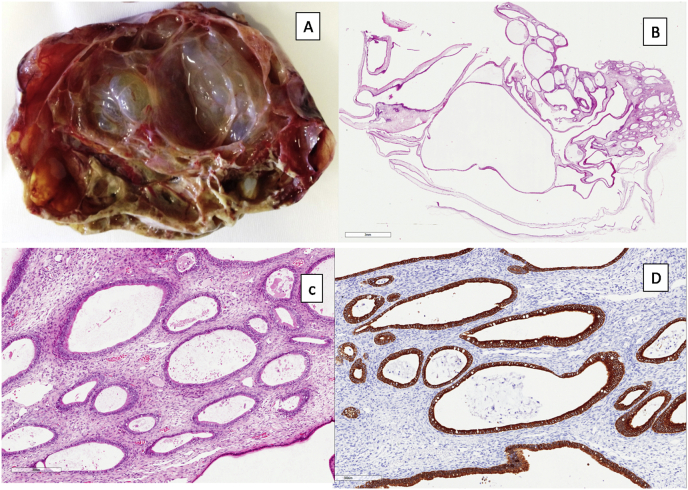
Histological examination. The ovary shows cystic formations with smooth outer and inner surfaces, some of which are filled with mucus (Panel A). Respectively at low (x10) and high (×40) magnification, dilated glands of different sizes with well-differentiated architecture, delimited from loose ovarian stroma (Panels B and C). Glandular epithelium of the ovary shows strong cytoplasmic positivity for CK19, compatible with a pancreatic-biliary metastatic neoplasm (Panel D).

A 82 years-old multiparous Caucasian woman was referred by family physician as she presented abdominal distension and pain and a palpable mass in the right iliac fossa. The past medical history included essential hypertension under pharmacological treatment and gastro-esophageal reflux. She denied any alcohol abuse or smoking. Blood tests showed CA 125 277 U/mL (reference range 0–36 U/mL) and CA 19.9 64.5 U/mL (reference range 0–37 U/ml). An abdominal ultrasonography and computed tomographic (CT) scan were obtained. Abdominal ultrasonography identified a heterogeneous, multi-septated mass with solid components occupying whole pelvis and a sclerotrophic gallbladder with wall thickening and biliary sludge. Abdominal CT-scan confirmed the thick-walled gallbladder close to the duodenum and the ovarian mass presenting hypodense areas with necrosis and calcifications with 17 × 11.5 cm in size, as shown in Fig. 1A–B. The patient, after careful anesthesiology evaluation, was considered fit for surgery. Explorative laparoscopy, performed by a senior surgeon with large expertise in laparoscopic surgery, identified an advanced gallbladder cancer with diffuse peritoneal carcinosis and a voluminous unilateral ovarian mass with initial signs of vascular distress. The other ovary was normal. Resection of the ovarian mass was performed and two peritoneal samples were sent for histologic examination. Peritoneal citology was positive for adenocarcinoma. Post-operative course was uneventful. Final histological examination of the ovarian tumor showed dilated glands of different sizes with well-differentiated architecture, delimited from loose ovarian stroma and the glandular epithelium presented strong cytoplasmic positivity for CK19, compatible with a pancreatic-biliary origin (Fig. 2A–D). The diagnosis of Krukenberg tumor by gallbladder cancer was done. The patient was discharged with a poor prognosis and she died three months after.

## 4 Discussion

Gallbladder carcinoma is a rare malignancy, but in selected areas of high incidence, such as India, Chile and Japan, it is a significant source of mortality [3]. The diagnosis in early stages is very hard because clinical symptoms are similar to those of benign disease, such as adenomyomatosis, acute or chronic cholecystitis, xanthogranulomatous cholecystitis, polyps and to other hepatobiliary malignancies, in particular intra-hepatic cholangiocarcinoma and hepatocellular carcinoma, in which there are specific clinical recommendations [3,[10], [11], [12]]. Most cases of gallbladder cancers are discovered accidently during surgical specimen's pathological examination after laparoscopic cholecystectomy. Different metastatic localizations by gallbladder carcinoma have been previously reported including liver (76–86%), lymph nodes (60%), adrenal glands, kidney, spleen, brain, breast, thyroid, skeletal system, heart and uterus [1,2].

The ovarian metastatic localization by biliary origin is a very uncommon finding in everyday clinical practice. Krukenberg tumor refers to a malignancy in the ovary that metastasized from a primary site, classically the gastrointestinal tract, although it can arise in other tissues. Gastric adenocarcinoma represents the most common source. Krukenberg tumours are often found in both ovaries, consistent with its metastatic nature. Krukenberg tumours are named after *Friedrich Ernst Krukenberg*, a German physician who reported what he thought was a new type of primary ovarian malignancy in 1896, a fibrosarcoma of the ovary [13]; six years later these were shown to be of metastatic gastrointestinal tract origin. In clinical practice, the differential diagnosis of an ovarian mass is quite hard, as approximately 7% of ovarian neoplasm encountered clinically are metastatic lesions, the most common sites of origin being the gastrointestinal tract. Metastases to female genital tract often pose diagnostic problems for both the clinicians and the pathologists. In a retrospective study over 147 patients, Li W et al. [14] reported a rate of ovarian metastases of 48.9% by colorectal cancer, 40.8% by gastric cancer, 8.2 by breast cancer and only 1.4% by biliary origin. Unfortunately, there are very few reports that describe cases of metastatic lesions that present clinically as a primary tumor [7,8,[15], [16], [17]]. The high rate of bilaterality, surface involvement by tumor cells, multinodular growth, extensive extra-ovarian tumor, size >10 cm, infiltrative and nodular pattern of invasion, and presence of signet ring cells are the most helpful features for indicating a metastatic nature of an ovarian mass [7,[18], [19], [20], [21]]. Kim SH et al. [20] also suggested that secondary ovarian neoplasm should be considered when solid ovarian tumours contain well-demarcated intraluminal cystic lesions. Seidman JD et al. [18] underlined the importance of tumor size for distinguishing primary and metastatic carcinomas in the ovary. According to him, all bilateral and unilateral carcinomas of diameter <10 cm are metastatic lesions, while unilateral carcinomas of diameter >10 cm can be considered as primary lesions. Conversely, in our case the patient presented a large unilateral ovarian mass of diameter >10 cm, which turned out to be a metastatic lesion.

Harcourt and Dennis [21] emphasized the need for complete pre-operative clinical evaluation in order to avoid unnecessary laparotomy for “ovarian cancer” which are in fact metastases from the colon. The prognosis of ovarian metastastes by biliary origin is very poor with an overall survival at around 6 months, as reported by Li W et al. [14]. Radiological investigations should help the clinicians to a better definition of the disease, but sometimes, such in the case described herein, can hide certain pitfalls [20]. The histopathological examination represents the most important tool for a correct diagnosis and immunohistochemistry plays a fundamental role [22,23]. There are no studies about therapeutic strategies and outcomes in ovarian metastases from biliary origin, because of its rarity and poor prognosis. Many studies have underlined the benefit of aggressive cytoreductive surgery and hyperthermic intraperitoneal chemotherapy (HIPEC) in Krukenberg tumours form gastric and colo-rectal cancers [16,17,21]. The identification of the primary tumor site is required to plan the best therapeutic option for these patients: sometimes, palliative surgery is required, as in this specific case.

## 5 Conclusion

The most important learning points are represented by the difficulty of clinical and radiological diagnosis of ovarian metastases, which may mimic a primary ovarian cancer, the enormous importance of histopathological evaluation and the need of multidisciplinary approach in order to avoid certain pitfalls. Indeed, the occurrence of Krukenberg tumor should be considered in the work-up of gallbladder cancer, by keeping in mind this rare occurrence in clinical practice.

## Ethical approval

This study is exempt from ethical approval in our institution.

## Sources of funding

This research did not receive any specific grant from funding agencies in the public, commercial, or not-for-profit sectors.

## Author contribution

Pesce A and La Greca G designed the paper, Pesce A performed review, Amore FF and Magro G performed histological analysis and provided histological pictures; Pesce A and Li Destri G wrote the paper, La Greca G and Puleo S supervised the paper; all the authors read and approved the final manuscript.

## Trial registry number

Not applicable. This case report is not the “*first case in man”.*

## Guarantor

On behalf of all authors, I am the guarantor who accept full responsibility for the work.

## Consent

Written informed consent was obtained from the patient for publication of this case report and accompanying images. A copy of the written consent is available for review by the Editor-in-Chief of this journal on request.

## Provenance and peer review

Not commissioned, externally peer reviewed.

## Declaration of competing interest

All the authors declare that they have no competing interests.
